# Effect of Admixtures on the Yield Stresses of Cement Pastes under High Hydrostatic Pressures

**DOI:** 10.3390/ma9030147

**Published:** 2016-03-02

**Authors:** Hong Jae Yim, Jae Hong Kim, Seung Hee Kwon

**Affiliations:** 1 Department of Construction and Disaster Prevention Engineering, Kyungpook National University, Sangju 37224, Korea; yimhongjae@gmail.com; 2 School of Urban and Environmental Engineering, Ulsan National Institute of Science and Technology, Ulsan 44919, Korea; 3 Department of Civil and Environmental Engineering, Myongji University, Yongin 17058, Korea; kwon08@mju.ac.kr

**Keywords:** pumped concrete, cement paste, high pressure, yield stress, rheology, admixture

## Abstract

When cement-based materials are transported at a construction site, they undergo high pressures during the pumping process. The rheological properties of the materials under such high pressures are unknown, and estimating the workability of the materials after pumping is a complex problem. Among various influential factors on the rheology of concrete, this study investigated the effect of mineral and chemical admixtures on the high-pressure rheology. A rheometer was fabricated that could measure the rheological properties while maintaining a high pressure to simulate the pumping process. The effects of superplasticizer, silica fume, nanoclay, fly ash, or ground granulated blast furnace slag were investigated when mixed with two control cement pastes. The water-to-cement ratios were 0.35 and 0.50.

## 1. Introduction

Large-scale construction, such as tall buildings, long-span bridges, and heavy infrastructures, is increasing; for efficiency, cement-based materials are frequently transported using pumping processes during construction [1,2,3,4,5,6]. Cement-based materials can be subjected to high pressures and flow rates during these pumping processes. For oil-well cementing applications, the pressure can reach 30 MPa. Evaluating the transporting efficiency of cement-based materials under high pressure is among the most important issues in the field of the large-scale construction.

Rheological assessment has been performed to evaluate the pumping efficiency of cement-based materials, and lubricating layers were considered to discuss flow-pumping pressure relationships [7]. A tribometer with a vane replaced by a cylinder was proposed, and several predictive models were developed to evaluate the rheological properties of such lubricating layers [3,4,7,8]. Recently, a full-scale pumping experiment was performed, and the rheological properties of the cement paste after pumping were measured and compared with those before pumping [4]. This test showed that the high pressure induced by pumping can decrease concrete viscosity and increase the yield stress [4]. It was also reported that a decrease in concrete slump flow after pumping occurred in the test, based on the long pipeline of 193 m [6]. These rheological changes under high pressure have been explained by several mechanisms: the fine and coarse aggregates may absorb free water from the added superplasticizer [1], the side chain length of a polycarboxylate superplasticizer may be reduced [9], and the separated and dispersed cement may induce a lack of water and superplasticizer by increasing the contact area between the cement particles and water [10]. However, another full-scale test, based on long vertical pipelines of 1 km, reported the conflicting result of increased slump flow after pumping [11]. These results indicate that the effects of high pressures on the rheological properties of cement-based materials are difficult to conclude, despite the data from several field tests.

Segmenting the complex and interrelated effect of materials on the pumping process may provide a better understanding of the rheological behavior of pumped concrete. This study concentrates experimental experience on the effects of admixture for cement-based materials. The use of chemical and mineral admixtures, such as the aforementioned superplasticizer, are indispensable for high-workability concrete. Chemical admixtures can change the shear field of concrete under the pumping process [12]. Shearing flow, similar to Poiseuille flow, describes the pumping flow of self-consolidating concrete, whereas the plug flow of conventional concrete is dominant on a lubricating layer of the inner surface of the pipeline. The use of chemical and/or mineral admixtures clearly increases the consistency of the self-consolidating concrete. However, the effects of these materials on the rheology of the lubricating layer, induced under high pumping pressures, are still unknown. Therefore, a series of experiments was conducted to quantify the effects of various admixtures on the increase in consistency of cement pastes under high (hydrostatic) pressures. The applied pressure levels corresponded to the ordinary level of pumping pressure. As a result, a dominant admixture was suggested to increase the consistency, and, therefore, the pumping efficiency of concrete.

## 2. Sample Preparation

A total of 91 cement paste samples were produced with ordinary Type I Portland cement and various types of admixtures. The specific gravity and specific surface area of the used cement were 3.14 and 3320 cm^2^/g, respectively. The specific surface area was determined using the Blaine test. Two types of superplasticizers were considered to produce high flow in the samples; these included polycarboxylate (PCE) and polynaphthalene sulfonate (PNS). For supplementary cementitious materials, fly ash (FA) and ground granulated blast-furnace slag (GGBFS) were used. The specific gravities of the FA and GGBFS were 2.44 and 2.95, respectively. Silica fume (SF), usually adopted for high-strength concrete, was also considered. Lastly, purified nanoclay (NC), geologically known as palygorskite or attapulgite, was also considered. Nanoclay particles have a needle-like shape, 1.8 μm length and 3.0 nm diameter; these are easily agglomerated on the microscale [13,14].

**Figure 1:**
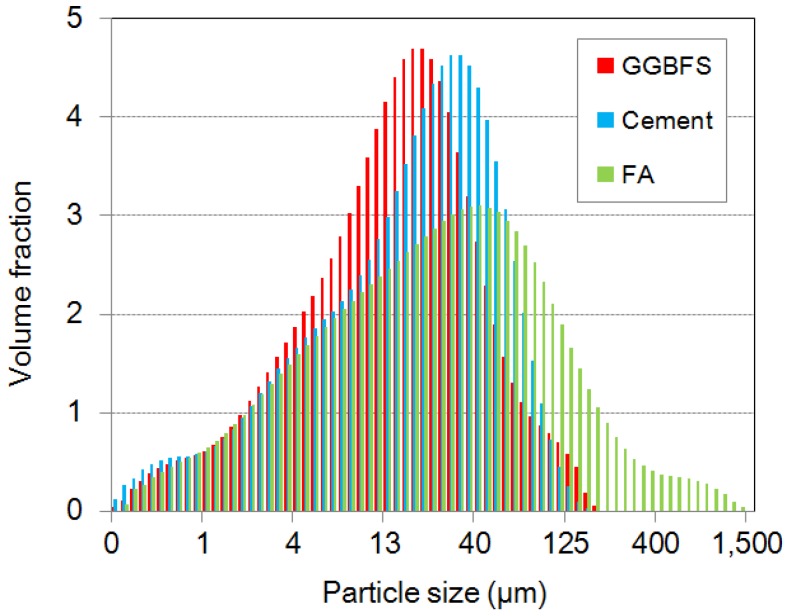
Measured particle size distribution of cement, ground granulated blast-furnace slag (GGBFS), and fly ash (FA).

The supplementary cementitious materials were measured by using a laser diffraction particle size analyzer. The obtained results are compared in logscale, as can be seen in Figure 1. The maximum size of cement and GGBFS is about 200 µm and FA is about 1500 µm. Even though the median of these powders is different, GGBFS and cement powders have similar size distributions and FA has a more broad distribution than other powders.

Table 1 reports the mix proportions of the samples. In the first group of prepared samples, WC35-PC0.0 is the control. The water-to-binder ratio (*w/b*) of the first group was 0.35 by mass. The compared samples incorporated PCE or PNS with dosages of 0.2% and 0.4% of the cement mass, respectively. The effects of SF or NC were compared in the second group, where the control sample was a cement paste, proportioned by *w/b* = 0.35 and a PCE dosage of 0.4%. The third and fourth groups were proposed to observe the effect of the replaced FA or GGBFS, where the replacement ratios by mass were 10%, 20%, or 30%, respectively. The effects of the admixtures were investigated in different *w/b*.

For each mixture, a 50 mL sample was produced. Each was mixed according to the following procedure to produce fresh-state samples: (1) hand-mixing for 0.5 min; (2) scraping the mixer for 2.5 min; and (3) hand-mixing again for 0.5 min. An additional 10 min was required to load the sample in a high-pressure rheometer. Finally, rheological measurements began at 13.5 min after the mixing was initiated. Room temperature (22 °C) and a relative humidity of 50% were maintained during the mixing and experiments.

## 3. Experimental Section

### 3.1. Instrument for Rheological Measurement under High Pressure

**Figure 2:**
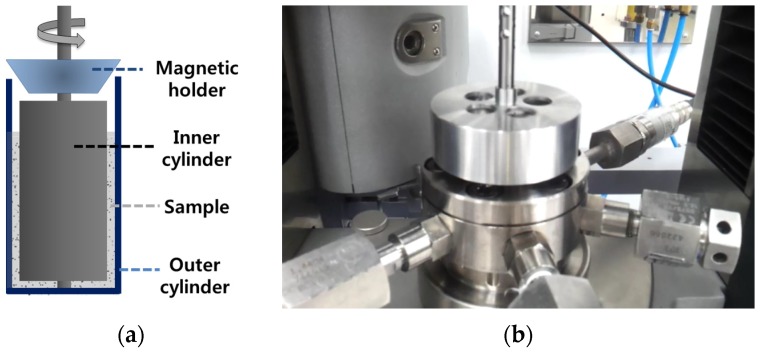
(**a**) Schematic of the high-pressure cell and (**b**) the setup launched in the rheometer.

A rheometer was fabricated to measure the rheological properties under high hydrostatic pressure. Figure 2 shows the schematic of the fabricated high-pressure cell, and the setup launched in the rheometer. Geometrically of the rheometer is a modified concentric cylinder, conventionally used for the rheological measurements. The gap size between the inner and outer cylinders is 2 mm, which is larger than the maximum size of the cement particles. The diameters of the inner and outer cylinders are 35 and 39 mm, respectively. The two cylinders were sealed using bolts, and then a high pressure was generated by filling the inner space with paraffin oil using a hand pump. The time used to apply the high pressure was approximately 15 min. The inner cylinder was then rotated to create a Couette flow in the samples, where the driving torque was transferred by a magnetic holder.

### 3.2. Rheological Measurements

The gravity-induced flow of concrete in the field (the slump flow) generally experiences a rate of shear strain of ~10 s^−1^. Simulating the pumping process and the shear field of the lubricating layer requires a much higher rate of shear strain. This study measured the flow curves of the prepared samples in a strain range from 10 to 1200 s^−1^, as the shear rate in the mockup pumping test exceeded 1000 s^−1^ [4]. A rate of 1200 s^−1^ was the maximum that could be generated by the fabricated rheometer.

**Figure 3:**
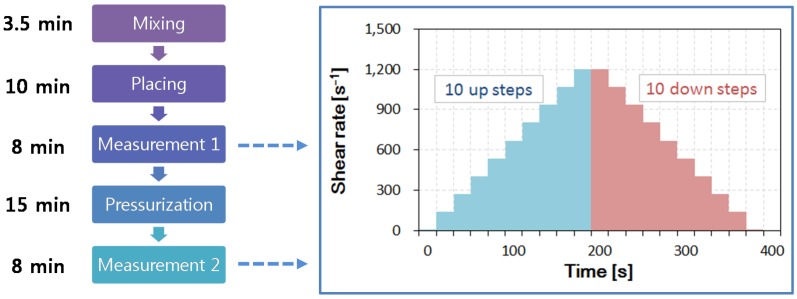
Timetable for measurement of yield stress under atmospheric pressure and high pressure, and its protocol to obtain a flow curve.

Figure 3 shows the timetable of the experimental procedure. A single procedure provides two flow curves. One depicts behavior under atmospheric pressure, and the other is obtained after the application of a high pressure. In this study, a rheological measurement under an atmospheric pressure (0 MPa in sealed pressure) is called “Measurement 1” and the one under high pressure (30 MPa) is “Measurement 2”. The intermediate pressure range, e.g., 10 and 20 MPa, was investigated in a preliminary test. The effects of high hydrostatic pressure on the flow curve was independent of the level of applied pressure. The flow curves in the range of 10 to 30 MPa were identical, and the variations of these curves, from those obtained under an atmospheric state, are of interest.

**Figure 4:**
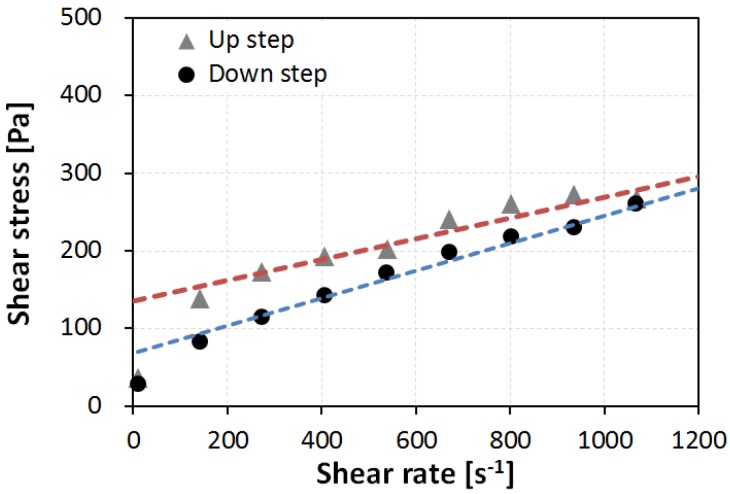
Obtained flow curves of the sample WB35-PCE0.2 according to the up step and down step of Measurement 1.

The protocol to obtain the two flow curves is the same as that shown in Figure 3. The up curve and the down curve were included in each flow curve obtained by 10 linear-scale steps, in which the strain rate was varied from 10 to 1200 s^−1^. In each linear step, the shear stress was measured over 20 s; the converged value of the shear stress was recorded at the rate of shear strain in each step. Figure 4 shows an example result from the WB35-PCE0.2 sample, where the up curve and down curve measurements are separated. The area between both curves indicates the thixotropy of the sample; it indicates the difference in the energy rate [15]. All prepared samples were thixotropic, meaning that the up curves were located above the down curves. The down curve is typically considered to determine the flow behavior, because the thixotropic effect can be excluded. This study also investigated the down curve to determine the effects of high pressure on the rheology.

**Figure 5:**
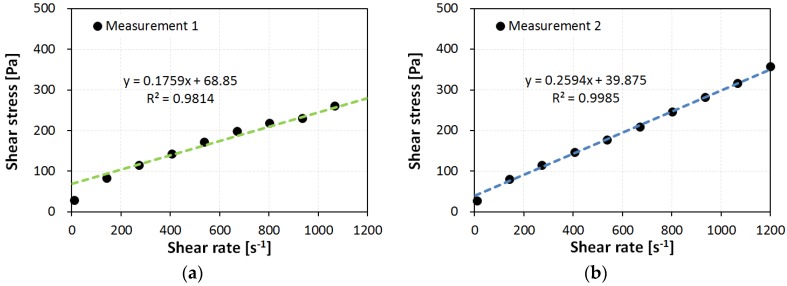
Down step for flow curve of sample WB35-PCE0.2 under atmospheric pressure; (**a**) Measurement 1 under atmospheric pressure and (**b**) Measurement 2 under a high pressure.

Figure 5 shows the down curves of sample WB35-PCE0.2, (a) under atmospheric pressure, Measurement 1; and (b) under high pressure, Measurement 2. The measured data points apparently follow a linear function with a non-zero *y*-intercept. Fitting these points using linear regression follow a Bingham-model fluid. The slope and *y*-intercept of the fitted linear function become the plastic viscosity η_p_ and yield stress τ_y_, respectively. As a result, each measurement can be represented by:

(1) $$\tau \left(\dot{\gamma }\right)=\tau_{y}+\eta_{p}\dot{\gamma }$$

where τ is the measured shear stress and $\dot{\gamma }$ is the applied rate of shear strain. In Figure 5, it is easily seen that the first point at 10 s^−1^ deviates from the linear trend. A previous study [16] reported that the first measured point usually included (1) transient fluctuations on the shearing of non-Newtonian fluids, which are systematic errors caused by the measuring geometry; and (2) thixotropic effects caused by the inherent characteristics of the sample material. Both are relevant to the experimental errors plausible in this investigation. Accordingly, excluding the first measured point for the model fitting allows a coefficient of determination of *R*^2^ > 0.95 for all samples.

## 4. Results and Discussion

### 4.1. Microstructural State

The high hydrostatic pressure supposedly changes the microstructure of the cement paste. The most promising hypothesis related to concrete pumping is that the pumping pressure accelerates the dispersion of the cement particles [10]. The coagulation of the cement particles diminishes and even vanishes in the presence of the high hydrostatic pressures of the interstitial fluid (water). No visual evidence to support this hypothesis has been reported, but the positive effect of the hydrostatic pressure on the consistency of the pumped concrete has been frequently reported.

The microstructural state of a suspension is discussed in terms of the fitting degree of the Bingham fluid model. The aforementioned *R*^2^ indicates the fitting performance for the Bingham fluid model. Higher values of *R*^2^ indicate a higher similarity between the cement paste sample and the Bingham fluid model. Among all test results, *R*^2^ under an atmospheric state was always less than that under a high-pressure state. For example, for the sample WB35-PCE0.2, in Figure 5, *R*^2^ were 0.9814 and 0.9985 for atmospheric and high pressures, respectively. The data points under atmospheric pressure were slightly off the linear line in comparison to those at high pressure.

**Figure 6:**
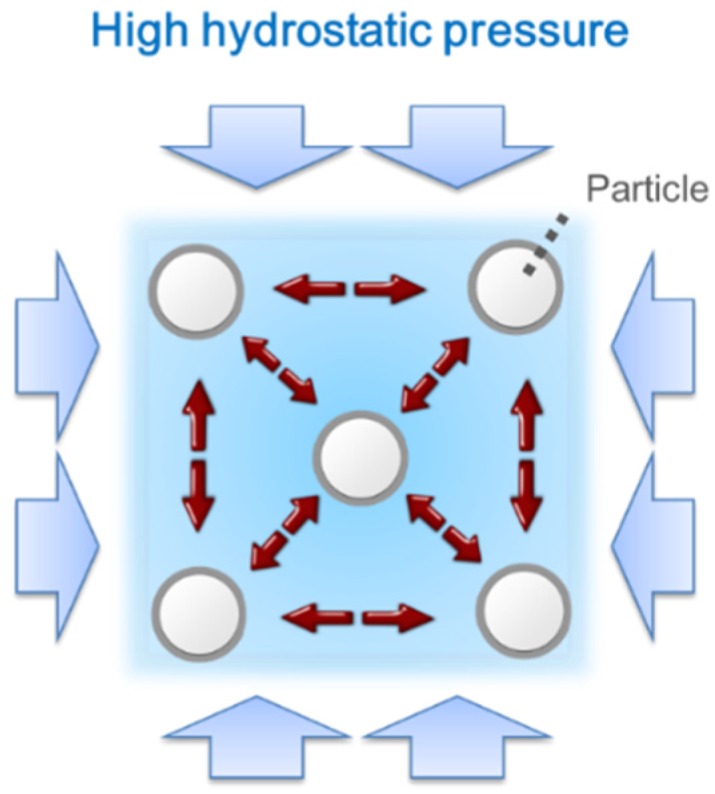
The repelling-particles system under high hydrostatic pressure.

On the other hand, the Krieger-Dougherty equation considers the interaction of particles in a very dense suspension [17], while the classical Einstein equation assumes there is no interaction of spherical particles in a diluted suspension (in which the solid volume fraction is below 10%). The particle interaction is described by the two parameters of packing density and the intrinsic viscosity of the particles. The intrinsic viscosity is approximately 6.0 for cement paste [18], and this value decreases by the incorporation of admixtures to reduce the viscosity. The hydrostatic pressure does not affect the drag force of a single particle; consequently, the flow resistance (intrinsic viscosity) of single particles is unchanged by high hydrostatic pressures. The packing state of the particles usually explains the change of a suspension’s microstructure under a certain intensity of shearing [19]. For example, a shear-thinning suspension is expected to have higher packing density at higher shear rates. The maximum packing density is close to 0.74, in which mono-sized spheres construct face-centered cubes. The density decreases with lower shearing rates. Therefore, a suspension shows a constant viscosity at all rates of shear strain only if the suspension has a consistent microstructure (and furthermore the packing density of particles) regardless of the intensity of shearing. The samples under high pressure showed a constant viscosity in which the applied shear stress exceeded the yield stress (high similarity to the Bingham fluid model), which indicates an increase in the packing stability of the cement particles. The high hydrostatic pressure in the interstitial fluid drives a repulsive force among the jammed particles. Figure 6 shows the state of repelling particles under high hydrostatic pressure. The particle interaction is consequently minimized at high pressure. In contrast, under atmospheric pressure, attraction prevails among the cement particles with the same interparticle distance. Cement agglomeration is dominant in this state [20].

### 4.2. Effect of Superplasticizer

**Figure 7:**
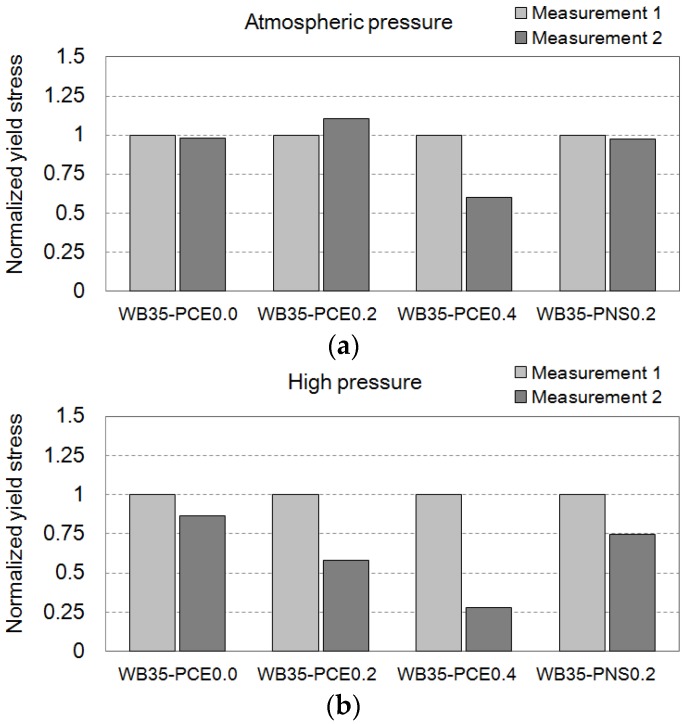
Normalized yield stress of the cement paste with different superplasticizers under (**a**) atmospheric pressure and (**b**) high pressure.

To understand the effect of chemical admixtures on the rheology of the cement pastes, comparative analyses of the obtained yield stresses were performed, as shown in Figure 7. The yield stresses are normalized according to the yield stress of Measurement 1 for each experiment. For example, replicated samples of WB35-PCE0.0 were measured twice: (1) Measurements 1 and 2 under atmospheric pressure; and (2) Measurement 1 under atmospheric pressure and Measurement 2 under high pressure. The result of Measurement 1 for each experiment was used as a control, and shows a variation within ±13 Pa, based on the average value of 116 Pa. Although this is an acceptable variation, considering the reproducibility of the rheological test of the cement pastes [16], the variation causes difficulties in establishing simple comparisons. In the bar charts of the normalized yield stresses, Measurements 1 and 2 can be intuitively compared according to the presence of high pressure. Under continuous atmospheric pressure, in Figure 7a, the differences between Measurements 1 and 2 are only the results of the 15 min delay in pressurizing the rheometer. This short delay in the pressurization hardly affects the yield stresses of the cement pastes. The effect of high pressure on the same sample ages can be seen in the second bar chart, Figure 7b.

The dosage of PCE is usually controlled to obtain concrete with a target consistency. The used PCE was effective when the dosage was 0.4% of the cement mass. In Figure 7a, the yield stress decreases with 0.4% PCE, while the samples WB35-PCE0.0 and WB35-PCE0.2 do not show PCE effects. Even in the mini-slump flow test, the 0.2% dosage was not effective. WB35-PCE0.2 showed 120 mm flow, but WB35-PCE0.4 showed 210 mm flow. Note that the bottom diameter of the mini-slump cone (Hagermann cone) was 100 mm. The high hydrostatic pressure activates the low-dosage PCE, and a decrease in the yield stress was found using WB35-PCE0.2. PCE polymers left in the interstitial water (most are absorbed by the cement particles) supposedly decrease the friction among the repelling particles under high hydrostatic pressures. Low dosages were ineffective when the cement particles showed high degrees of agglomeration. In the case of PNS, the high-pressure effect was valid. A 0.2% dosage was not effective at an atmospheric pressure. The mini-slump flow at this dosage was 120 mm. However, the yield stress was decreased when high pressure was applied.

The effect of PCE is generally described by the dispersion of the cement particles, which results in a decrease in the yield stress of cement paste. Several models were proposed to describe the relationship between the yield stress and the particle (agglomerate) size distribution [21,22]. A higher hydrostatic pressure in interstitial water supposedly deflocculates the cement suspension, and the consequent change of interparticle distance may reduce its yield stress. This is one of the possible mechanisms to support the increase in the consistency of pumped concrete. In addition, as introduced in the previous section, the repelling-particles system under high hydrostatic pressure increases the repulsive potential of the particles’ interactions, which also results in the decrease in its yield stress. Both hypotheses correspond with the current experimental results. Nevertheless, it is hard to conclude which effect is more dominant.

### 4.3. Effect of Nanoclay and Silica Fume

**Figure 8:**
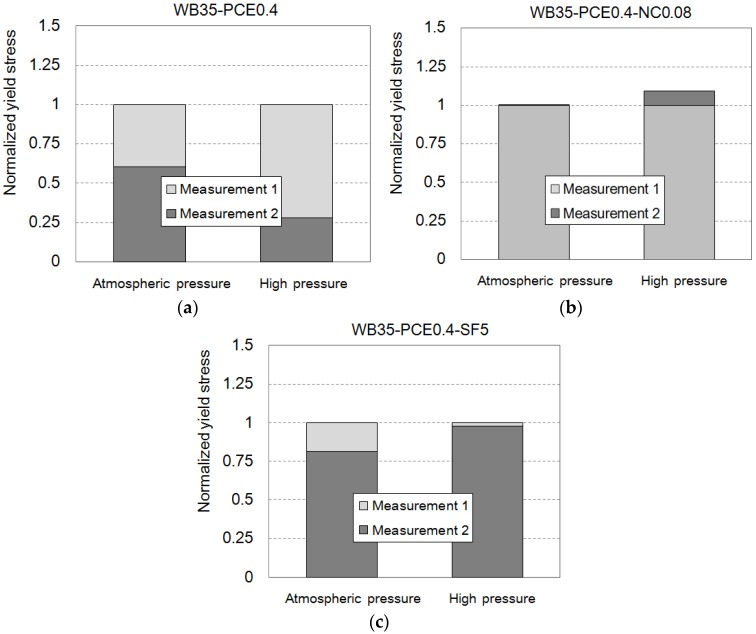
Normalized yield stress of the cement paste with (**a**) superplasticizer; (**b**) nanoclay; and (**c**) silica fume under atmospheric pressure and high pressure.

Silica fume (SF) reportedly decreases the consistency of cement-based materials [23], and nanoclay (NC) increases the thixotropy of cement-based materials [14,24]. Both require much lower dosages to achieve a target consistency than superplasticizers. In this study, a PCE dosage of 0.4% was considered in cement paste. Under atmospheric pressure, 0.08% NC addition by cement mass created the effect of 0.4% PCE, as shown in Figure 8a. Five percent SF addition also increases the yield stress. These effects of NC and SF were enhanced under high pressure, as can be seen in Figure 8b. The fineness of SF and NC provide high degrees of water adsorption, which may be the source of the effect on the rheology of the cement [14]. High hydrostatic pressure increases the capability of the admixtures for water adsorption. Therefore, NC and SF should be used cautiously for pumped concrete.

### 4.4. Effect of Supplementary Cementitious Materials

**Figure 9:**
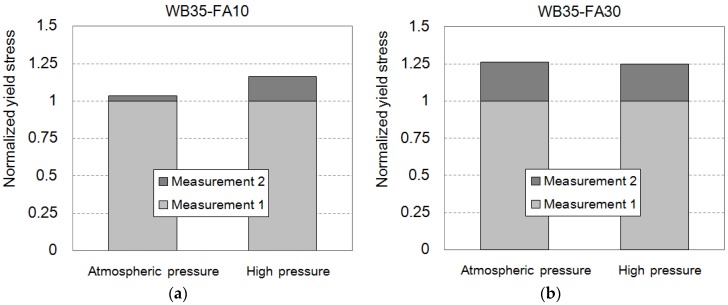
Normalized yield stress of the cement paste with different fly ash replacement of (**a**) 10% and (**b**) 30% under atmospheric pressure and high pressure.

Generally, FA replacement is reported to reduce the yield stress and the viscosity of cementitious paste. FA particles are spherical and act much like ball bearings in suspensions [23]. Another explanation for FA consistency enhancement is the dilution of the cement paste, which results in a decrease of the yield stress [25]. The yield stresses of Measurement 1 were averaged with replicated samples: 82.9 Pa for WB35-FA10, 72.5 Pa for WB35-FA20, and 59.4 Pa for WB35-FA30. Kashani *et al.* provide a model to predict the yield stress using width parameter of particle size distribution and mass ratio of used powder [22]. As can be seen in Figure 1, used FA has a more broad particle size distribution than other powders used, and it leads to the decrease of yield stress from 114.8 Pa of cement paste without any admixture (WB35-PCE0.0) to mixes with replaced FA. Increasing the replacement ratio decreases the yield stress, while the changes in the plastic viscosity are marginal (0.243, 0.256 and 0.326 Pa∙s, respectively). Very high rates of shear strain of 1200 s^−1^ weaken the ball-bearing or diluting effect, because, at these rates, the collisional effects of the particles are dominant with a high Bagnold’s number [26]. The interaction of particles is less influential. Consequently, the enhanced consistency by FA is weakly observed. Additional measurements under atmospheric pressure show a slight increase in yield stress, as shown in Figure 9a. Increasing the collision frequency by additional shearing is thought to contribute to the increase in yield stress.

The high hydrostatic pressure impedes the increase in yield stress, as shown in Figure 9b. The collision frequency is further increased by the state of the repelling particles. Therefore, FA replacement is expected to negatively affect pumping performance, which corresponds to the results of mockup tests. In a previous study [4], 10% FA replacement decreased the flow rate by approximately 10% to 15% in a 300-m pumping test.

GGBFS-cement pastes were also tested to investigate the rheological behavior of the material under high shear rate and hydrostatic pressure. However, it was difficult to determine trends in the effects of GGBFS. The yield stresses and plastic viscosities of the GGBFS-cement pastes did not show noticeable trends. A mixture of pulverized particles of Portland cement and GGBFS shows no particular effects compared to the control cement paste. In the case of FA, the shape of the replaced particles differed. Incorporating GGBFS does not change the rheology when the effect of the particle interaction of particles is weak under high shear rates or high pressures. In contrast with FA, the replaced GGBFS induces a decrease in the yield stress at atmospheric pressure. This effect is not determined by the replacement ratio of GGBFS, but by affecting the water to powder ratio, which is greater with larger water contents in the mixture. This slag effect is also seen under high pressure, but only in the mixture with a larger amount of water *w/b* = 0.5. In the mixture with *w/b* = 0.35, the replaced GGBFS induces an increase in the yield stress under high pressure. This appears to result from the fact that a large amount of water in the mixture mainly affects the yield stress, rather than the use of GGBFS.

## 5. Conclusions

This study attempted to investigate the effects of admixtures on the rheology of cementitious materials and, ultimately, to evaluate the pumping performance of concretes with these admixtures. The rheological measurements of various samples were performed under high hydrostatic pressure. The rate of shear strain was specially selected in the range from 10 to 1200 s^−1^ to simulate the pumping process. The measured rheology was dependent on the presence of a high hydrostatic pressure. The test pressure exceeded 10 MPa. This high pressure induced a state of repelling particles, in which the repulsive interparticle forces dominated in all tested cementitious suspensions. The repelling-particle system revealed the effect of superplasticizers at relatively low dosages and increased the capacity for water absorption of the nanosized particles. The use of silica fume or nanoclay requires care for pumped concrete. The use of fly ash negatively affects pumping ability, as it decreases the consistency of the cementitious paste in the pumped concrete.

## Figures and Tables

**Table 1:** Mix proportion of the cement paste with different admixtures.

Label	*w*/*b* [%]	Superplasticizer [%]	Nanoclay [%]	Silica Fume [%]	Fly Ash [%]	GGBFS [%]
WB35-PCE0.0	35	-	-	-	-	-
WB35-PCE0.2	35	0.2 (PCE)	-	-	-	-
WB35-PCE0.4	35	0.4 (PCE)	-	-	-	-
WB35-PNS0.2	35	0.2 (PNS)	-	-	-	-
WB35-PCE0.4	35	0.4 (PCE)	-	-	-	-
WB35-PCE0.4-NC0.08	35	0.4 (PCE)	0.08	-	-	-
WB35-PCE0.4-SF5	35	0.4 (PCE)	-	5	-	-
WB35-FA10	35	-	-	-	10	-
WB35-FA20	35	-	-	-	20	-
WB35-FA30	35	-	-	-	30	-
WB35-BS10	35	-	-	-	-	10
WB35-BS20	35	-	-	-	-	20
WB35-BS30	35	-	-	-	-	30
